# Pressure Overload Is Associated With Low Levels of Troponin I and Myosin Binding Protein C Phosphorylation in the Hearts of Patients With Aortic Stenosis

**DOI:** 10.3389/fphys.2020.00241

**Published:** 2020-03-19

**Authors:** O’neal Copeland, Andrew Messer, Andrew Jabbour, Corrado Poggesi, Sanjay Prasad, Steven Marston

**Affiliations:** ^1^Imperial College London, London, United Kingdom; ^2^Royal Brompton Hospital, and Imperial College London, London, United Kingdom; ^3^University of Florence, Florence, Italy

**Keywords:** troponin I, phosphorylation, aortic stenosis, pressure overload, cardiomyopathy, protein kinase A, myosin binding protein C

## Abstract

In previous studies of septal heart muscle from HCM patients with hypertrophic obstructive cardiomyopathy (HOCM, LVOT gradient 50–120 mmHg) we found that the level of phosphorylation of troponin I (TnI) and myosin binding protein C (MyBP-C) was extremely low yet samples from hearts with HCM or DCM mutations that did not have pressure overload were similar to donor heart controls. We therefore investigated heart muscle samples taken from patients undergoing valve replacement for aortic stenosis, since they have pressure overload that is unrelated to inherited cardiomyopathy. Thirteen muscle samples from septum and from free wall were analyzed (LVOT gradients 30–100 mmHg) The levels of TnI and MyBP-C phosphorylation were determined in muscle myofibrils by separating phosphospecies using phosphate affinity SDS-PAGE and detecting with TnI and MyBP-C specific antibodies. TnI was predominantly monophosphorylated and total phosphorylation was 0.85 ± 0.03 molsPi/mol TnI. This phosphorylation level was significantly different (*p* < 0.0001) from both donor heart TnI (1.6 ± 0.06 molsPi/mol TnI) and HOCM heart TnI (0.19 ± 0.04 molsPi/mol TnI). MyBP-C is phosphorylated at up to four sites. In donor heart the 4P and 3P species predominate but in the pressure overload samples the 4P species was much reduced and 3P and 1P species predominated. Total phosphorylation was 2.0 ± 0.2 molsPi/mol MyBP-C (*n* = 8) compared with 3.4 ± 0.07 (*n* = 21) in donor heart and 1.1 ± 0.1 (*n* = 10) in HOCM heart. We conclude that pressure overload may be associated with substantial dephosphorylation of troponin I and MyBP-C.

## Introduction

Cardiac muscle contractility is modulated by the β-adrenergic system that primarily acts via activation of protein kinase A (PKA). In the cardiac muscle sarcomere the main targets of PKA are myosin binding protein C (MyBP-C) and Troponin I (TnI) and phosphorylation of these proteins plays a vital role in the enhanced contraction and relaxation kinetics induced by adrenergic stimulation (Layland et al., 2005b; Barefield and Sadayappan, 2010; Messer and Marston, 2014). In non-diseased heart muscle from donor human hearts or from mice, the level of phosphorylation of TnI and MyBP-C are both relatively high (Messer et al., 2009; Copeland et al., 2010). TnI has a total level of phosphorylation of 1.6 molsPi/mol in human and 1.2 in mouse with the majority of TnI being bis-phosphorylated. MyBP-C has a total level of phosphorylation of 3.4 molsPi/mol MyBP-C with 4P and 3P species predominating.

In heart disease the phosphorylation level is often low. For instance in idiopathic (non-ischaemic) end stage heart failure explants, phosphorylation levels are 0.26 molsPi/mol TnI and 0.62 molsPi/mol MyBP-C (van der Velden et al., 2003; Messer et al., 2007; Zaremba et al., 2007; Messer et al., 2009). In genetic heart disease the situation appears to be more complex. Explanted heart samples with inherited DCM can be associated with high or low levels of phosphorylation in the range 0.3–1.5 molsPi/mol TnI (Memo et al., 2013). However, myectomy samples taken from patients with inherited hypertrophic obstructive cardiomyopathy (HOCM) always have a low level of phosphorylation (Messer et al., 2009; Copeland et al., 2010; Bayliss et al., 2012). Thus, there appears to be no direct relationship between mutations causing heart disease and the TnI and MyBP-C phosphorylation level.

The HCM-associated mutations often cause a hypertrophied interventricular septum that can lead to left ventricular outflow tract obstruction (LVOTO). The septal myectomy operation for patients with HCM is usually indicated to reduce the obstruction when there is a high Aorta/LV pressure difference, typically 100 mmHg (Firoozi et al., 2002; Elliott and McKenna, 2004). We hypothesized that the pressure gradient may be a major factor in inducing the secondary phenotype of HOCM heart. To test this we have studied TnI and MyBP-C phosphorylation levels in heart muscle from patients with HCM but without pressure overload and heart muscle from patients without HCM but with pressure overload due to aortic stenosis (Bates, 2011).

## Methods

### Tissue Sources

Donor hearts and the K280N HCM sample were supplied by the Sydney Heart Bank (Li et al., 2013; Messer et al., 2016). Donor sample (NH) had no history of cardiac disease and normal ECG and ventricular function and were obtained when no suitable transplant recipient was found. HOCM sample (MV) was obtained from a patient undergoing septal myectomy operation at The Heart Hospital (UCL), London (Jacques et al., 2008). Clinical data for NH and MV has been previously reported in Messer et al. (2007), Bayliss et al. (2012) and in Supplementary Table 1. Measurements of TnI and MyBP-C phosphorylation were described in Messer et al. (2009) and Copeland et al. (2010).

Biopsies were taken from septum and free wall of patients undergoing valve replacement surgery to relieve aortic stenosis at the Royal Brompton Hospital, London and Careggi University Hospital, Florence. The ACTC E99K patient sample was kindly supplied by Dr. Lorenzo Monserrat, La Coruña, Spain (Monserrat et al., 2007; Song et al., 2011). Available clinical data on these samples is given in the Supplementary Material.

Patients gave written consent with PIS approved by the relevant ethical committee. All samples are anonymised. The investigations conform to the principles of the Declaration of Helsinki.

Ethical approval for collection and distribution of the human heart samples was granted by the Research Integrity, Human Research Ethics Committee, University of Sydney (Protocol No. 15401); the Joint UCL/UCLH Ethics Committee Rec No: 04/0035; Outer North East London Research Ethics Committee REC ref: 10/H0701/8; Careggi University Hospital Ref: 2006/0024713 and Comite Ético de Investigatio ń Clìnica de Galicia. Permission for study of the samples was granted by the NHS National Research Ethics Service, South West London REC3 (10/H0803/147).

### Measurement of TnI and MyBP-C Phosphorylation Level

Twenty milligrams of heart muscle sample, stored in liquid nitrogen were crushed in a liquid nitrogen cooled percussion mortar and then homogenized in 200 μl buffer (5 mmol/l NaH_2_PO_4_, 5 mmol/l Na_2_HPO_4_ pH 7.0, 0.1 mol/l NaCl, 5 mmol/l MgCl_2_, 0.5 mmol/l EGTA, 0.1% Triton X-100, 20 mmol/l NaF and 5 mmol/l DTT with 2 μg/ml each of the protease inhibitors E64, chymostatin, leupeptin and pepstatin A). The homogenate was then centrifuged at 16,500 × *g* for 5 min and the supernatant discarded. The wash process was repeated and then the pellet was dissolved in sample buffer containing 8 M urea, 2 M thiourea, 0.05 M Tris-HCl, pH 6.8, 75 mM DTT, 3% SDS and 0.05% bromophenol blue as decribed (Layland et al., 2005a; Messer et al., 2007).

TnI phosphorylation levels in heart muscle myofirils was measured by Phosphate affinity SDS-PAGE as described by Messer et al. (2009). Discontinuous SDS-PAGE gels were hand-cast and run using the Mini-PROTEAN system (Bio-Rad). Gel compositions are as follows: stacking gel: 4% acrylamide (29:1 acrylamide:bis-acrylamide), resolving gel: 10% acrylamide (29:1 acrylamide:bis-acrylamide, 100 μM MnCl_2_ (from 10 mM stock) and 50 μM Phos-TagTM acrylamide [from 5 mM stock solution of Phos-TagTM acylamide AAL-107 (NARD Institute, Hyogo, Japan)] prepared according to suppliers instructions (Kinoshita et al., 2006).

Gels were probed with the phosphorylation-independent anti-human-cardiac troponin I (hcTnI) clone 14G5 mouse mAb (Abcam plc antibodies), 1/2,000 dilution on Western blots. Secondary antibody was HRP- conjugated anti-rabbit IgG (1:1,000) and the blots were visualized using ECL (GE Biosciences). Chemiluminescence was detected by a cooled CCD camera-based gel imager (G:BOX Chemi HR16, Syngene).

To resolve MyBP-C phosphospecies the myofibril samples were run on the gels for 165 min. The current was initially 25 mA, raised to 35 mA once the samples had entered the resolving gel. The gels were Western blotted and probed with a rabbit polyclonal antibody against cMyBP-C residues 2–14 which recognizes total cMyBP-C or with phosphorylation site-specific antibodies (Bardswell et al., 2009; Sadayappan et al., 2009; Copeland et al., 2010).

## Results and Discussion

### Reduced Level of Phosphorylation in Pressure Overloaded Heart

We studied 13 heart muscle biopsies from intraventricular septum and free wall taken from patients undergoing valve surgery to relieve pressure overload and compared them with previously studied donor heart samples and myectomy samples from patients with HOCM (Messer et al., 2009; Copeland et al., 2010). LVOT gradients ranged from 30 to 100 mmHg in the pressure overload patients compared with 90–120 mmHg in the HOCM patients’ hearts and close to zero in the donor hearts (see Supplementary Tables 1 and  2).

**FIGURE 1:**
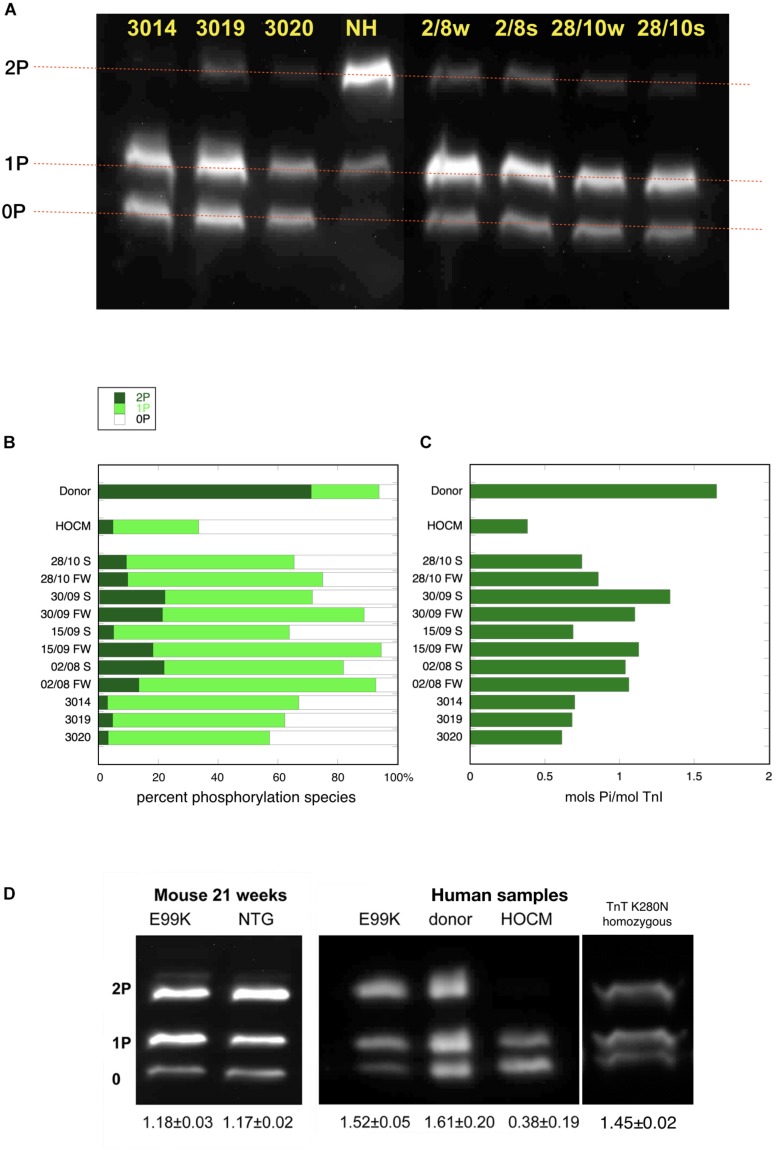
Phosphate affinity SDS-PAGE separation of myofibrils, probed with antibody 14G5 to troponin I. **(A)** Example of separation of phosphospecies. Phosphorylated protein is retarded in proportion to level of phosphorylation yielding discrete bands for bis- mono- and unphosphorylated troponin I. NH is a donor heart sample showing a high level of 2P species. The other lanes are aortic stenosis samples which show high level of 1P and 0P species. **(B)** Distribution of 2P, 1P, and 0P species in heart muscle samples. The results for each sample are the means of 2–4 replicate assays. S, septum; FW, free wall. Donor heart NH and HOCM heart MV control results are the mean of replicates included in the same gels as the pressure overload samples. Full data is shown in Supplementary Table 2. **(C)** Calculated total phosphorylation level for these samples. **(D)** Phosphate affinity SDS-PAGE of ACTC E99K mouse, ACTC E99K human heart and TNNT2 K280N human heart samples with donor and HOCM controls. Total phosphorylation is shown below underneath each lane.

The levels of TnI and MyBP-C phosphorylation were determined in muscle myofibrils by separating phosphospecies using phosphate affinity SDS-PAGE and detecting with TnI and MyBP-C specific, but phosphorylation-independent antibodies previously characterized. This technique measures the proportions of bis-phosphorylated, monophosphorylated and unphosphorylated species of TnI. We previously showed that in donor hearts, 70% of the troponin I is bis-phosphorylated and 21% is monophosphorylated with a calculated total phosphorylation of 1.6 ± 0.06 molsPi/mol TnI. The HOCM samples were just 5% bis-phosphorylated and 30% monophosphorylated with a calculated total phosphorylation level of 0.18 ± 0.02 molsPi/mol TnI (Messer et al., 2009; Figure 1).

In the pressure overload samples, we found that TnI was predominantly monophosphorylated (0P = 24 ± 2%, 1P = 67 ± 2%, 2P = 9 ± 1%, *n* = 21) and total phosphorylation was 0.85 ± 0.03 molsPi/mol TnI and was not significantly different in septum and free wall samples (see Supplementary Figure 1). Thus in pressure overload samples, the phosphorylation level was significantly less than donor heart and significantly more than in HOCM heart Troponin I (*p* ≤ 0.0001). Western blotting using antibodies to bis-phosphorylated troponin I confirmed that phosphorylation at serines 22 and 23 was low relative to donor heart samples. Mass spectrometry measurements showed that only Ser22 or Ser23 were phosphorylated with no evidence for phosphorylation at other sites in Troponin I.

**FIGURE 2:**
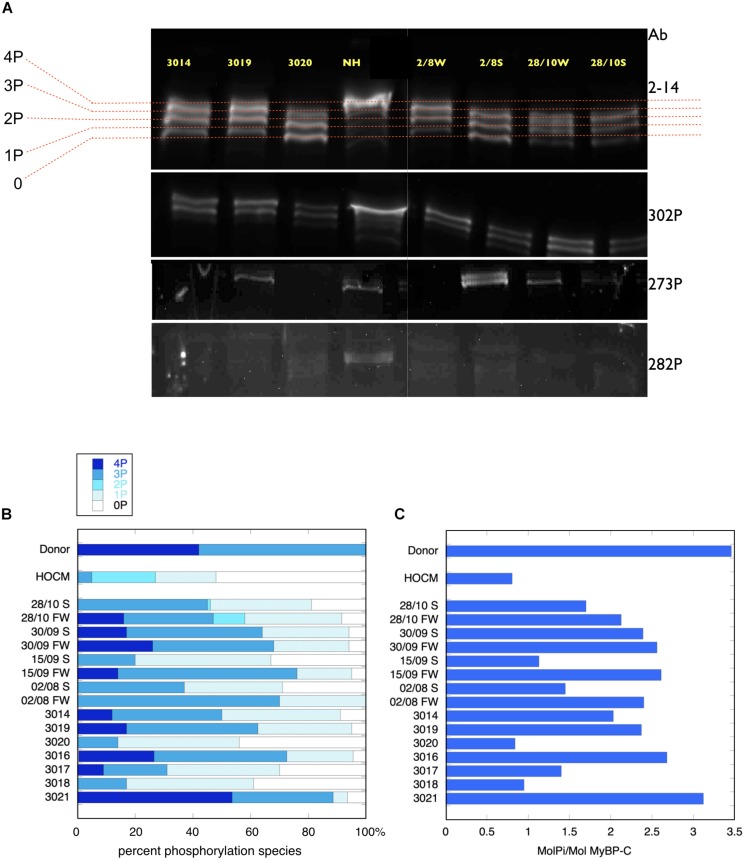
Phosphate affinity SDS-PAGE separation of myofibrils, probed with antibodies to MyBP-C. (**A**, top) Example of separation of MyBP-C phosphospecies probed with 2–14 pan MyBP-C antibody. NH is a donor heart sample showing a high level of 3P and 4P species. The other lanes are aortic stenosis samples which show less 4P and more 1P and 0P species. (**A**, lower panels) the same samples probed with antibody specific to phosphorylated ser 302, ser 273 and ser 282. **(B)** Distribution of 4P, 3P, 2P, 1P, and 0P species of MyBP-C in heart muscle samples. The results for each sample are the means of 2–4 replicate assays. Donor heart NM and HOCM heart MV control results are the mean of replicates included in the same gels as the pressure overload samples. Full data is shown in Supplementary Table 3. S, septum; FW, free wall. **(C)** Calculated total phosphorylation level for these samples.

MyBP-C is phosphorylated at up to four sites. In donor heart samples we showed that the 4P and 3P species predominate (4P = 43%, 3P = 57%, 2P = 0%, 1P = 0%, 0P = 0%) (Copeland et al., 2010). In the pressure overload samples the 4P species was much reduced and 3P and 1P species predominated (4P = 9 ± 3%, 3P = 41 ± 5%, 2P = 1 ± 1%, 1P = 33 ± 2%, 0P 15 ± 4%, *n* = 15) (Figure 2). Total phosphorylation was 2.0 ± 0.2 molsPi/mol MyBP-C (*n* = 15) compared with 3.4 ± 0.07 (*n* = 21) in donor heart and 1.1 ± 0.1 (*n* = 10) in HOCM heart (Copeland et al., 2010). Comparison of free wall and septal samples indicated higher phosphorylation levels in the free wall that was barely statistically significant (see Supplementary Figure 2). We used antibodies specific to the main targets of PKA in MYBP-C: phosphorylation sites at Ser 302, 273 and 283 but found no consistent pattern of phosphorylation in the pressure overload samples that was different from the pan-MyBP-C antibody, 2–14.

### Normal Level of Phosphorylation in HCM Samples Without Pressure Overload

To test whether the low level of phosphorylation in HOCM myectomy samples is due to the mutation or the pressure overload, we investigated samples from HCM patients that did not have pressure overload. The ACTC E99K mutation is associated with HCM, with the hypertrophy often confined to the apex of the heart and does not develop HOCM (Monserrat et al., 2007). We have previously studied a biopsy from a 33 year old patient with the ACTC E99K mutation who showed no signs of LVOTO and was not on any medication (Song et al., 2011). The tissue sample was obtained from an operation to repair an atrial septal defect. Troponin I, troponin T, and MyBP-C phosphorylation levels in myofibrils were the same as donor heart samples (see Figure 1D). Likewise, *The ACTC* E99K mouse model of HCM develops apical hypertrophy without LVOTO or symptoms of heart failure at 21 weeks (Song et al., 2011). The HCM mouse myofibrils have the same level of troponin I, troponin T, and MyBP-C phosphorylation as NTG littermates (Figure 1D).

We have also studied a heart muscle sample from a patient with an HCM-causing mutation [homozygous TNNT2 K280N (Sequeira et al., 2013; Messer et al., 2016; Piroddi et al., 2019)]. The patient had a myectomy operation that relieved LVOTO permanently but later developed heart failure requiring a heart transplant. This sample is from the explanted heart. The troponin I phosphorylation level was comparable to donor heart (Figure 1D). These two examples suggest that reduced levels of phosphorylation are related primarily to the pressure overload.

## Conclusion

In these limited studies on human heart muscle samples we have found that a reduced level of phosphorylation of TnI and MyBP-C, the sarcomeric targets of PKA, is associated with pressure overload, i.e., a mean transvalvular pressure gradient >40 mmHg, but is not associated with hypertrophic cardiomyopathy in the absence of pressure overload.

Since we are using human samples obtained during surgery our results could be confounded by a number of uncontrolled variables such as the time between excision of the sample and freezing and the medication, particularly beta-blockers, taken by the patients. On the other hand we have more than 12 years experience making measurements with human heart samples and have not yet been able to detect a correlation between these variables and any measurements we made (Messer et al., 2007, 2009; Jacques et al., 2008; Copeland et al., 2010; Song et al., 2011; Bayliss et al., 2012; Memo et al., 2013).

We do not know the underlying cause of the reduced phosphorylation levels but in HCM and heart failure there is evidence for both the reduction in PKA activity and the increase in phosphatase activity in pathological heart muscle that could account for this observation. In the diseased myocardium β adrenoceptors are often downregulated via receptor phosphorylation and β-arrestin binding (Mangmool et al., 2018) whilst protein phosphatase activity is enhanced via inactivation of phosphatase inhibitor-1 (El-Armouche et al., 2004; Champion, 2005).

## Data Availability Statement

All datasets generated for this study are included in the article/Supplementary Material.

## Ethics Statement

Ethical approval for collection and distribution of the human heart samples was granted by the Research Integrity, Human Research Ethics Committee, University of Sydney (Protocol No. 15401); the Joint UCL/UCLH Ethics Committee Rec No: 04/0035; Outer North East London Research Ethics Committee REC ref: 10/H0701/8; Careggi University Hospital Ref: 2006/0024713 and Comite É t́ico de Investigatio ń Clínica de Galicia. Permission for study of the samples was granted by the NHS National Research Ethics Service, South West London REC3 (10/H0803/147). The patients/participants provided their written informed consent to participate in this study.

## Author Contributions

OC, AM, and SM performed the experimental work. AJ, CP, and SP supplied the tissue samples. All the authors contributed to and approved the final manuscript that was edited by SM.

## Conflict of Interest

The authors declare that the research was conducted in the absence of any commercial or financial relationships that could be construed as a potential conflict of interest.
