# Supplementary material for: Pressure Overload Is Associated With Low Levels of Troponin I and Myosin Binding Protein C Phosphorylation in the Hearts of Patients With Aortic Stenosis
Source: Front Physiol. 2020 Mar 19;11:241. doi: 10.3389/fphys.2020.00241 (PMC7096377; doi:10.3389/fphys.2020.00241)
Supplement: Supplementary file 1 [file Table_1.DOCX]

SUPPLEMENTARY DATA

**Pressure overload is associated with low levels of Troponin I and Myosin binding protein C phosphorylation in the hearts of patients with aortic stenosis.**

O’neal Copeland^1^, Andrew Messer^1^, Andrew Jabbour^2^, Corrado Poggesi^3^, Sanjay Prasad ^2^, Steven Marston^1^

**Table 1**

**CLINICAL DATA ON PRESSURE OVERLOAD SAMPLES:**

**FI 3014**: Male 76y, severe mitral prolapse and mitral insufficiency (non-severe secondary hypertrophy).

**FI3016**: Female 70yy, Aortic V Stenosis; EF 50%, IVS thickness 14 mm, V/Ao gradient 125 mmHg (max)

**FI 3017**: Male 40y, Severe Aortic V stenosis (Rheumatic); EF 60%, IVS thickness 15 mm, V/Ao gradient 115 mmHg (max) 70 mmHg (mean)

**FI 3018**: Female 64y, Moderate/Severe Aortic V Stenosis; EF 59%, IVS thickness 15 mm, V/Ao gradient 45 mmHg (max) 28 mmHg (mean)

**FI3019**: Female 76y, Aortic V stenosis; IVS thickness 14 mm, V/Ao gradient 56 mmHg (max) 30 mmHg (mean)

**FI3020**: Female 74y, Aortic V stenosis; IVS thickness 14 mm, V/Ao gradient 61 mmHg (max)

**FI 3021**: Male 66y, Severe Aortic V Stenosis; EF 50%, IVS thickness, 13 mm, V/Ao gradient 49 mmHg (mean)

**NH:** Female 48y, subarachnoid haemorrhage

**MV:** Male 53y, Familial hypertrophic cardiomyopathy, MyBP-C truncating mutation IVS17+5G>T, resting V/Ao gradient 105 mmHg, IVS thickness 24 mm

__________________________________________________________________

**Supp Table 2**

Complete results for TnI phosphorylation measurements

**Supp Table 3**

Complete results for MyBP-C phosphorylation measurements

**Supp Figure 1**

Comparison of troponin I phosphorylation level in septum and free wall of pressure overload samples

Mols P/

Mol TnI

Eight samples were available from free wall and septum of the same heart. Their phosphorylation level is plotted here. In a paired t-test, p=0.54 whilst in an unpaired t test p=0.53 indicating the difference is not significant.

Free wall samples n=20 measurements, mean phosphorylation level is 0.93±0.17 (SEM)

Septum samples n=19 measurements, mean phosphorylation level is 0.85±0.06

All samples n=35 measurements, mean phosphorylation level is 0.88±0.04

**Supp Figure 2**

Comparison of MyBP-C phosphorylation level in septum and free wall of pressure overload samples

Mols P/

Mol MyBP-C

Four samples were available from free wall and septum of the same heart. Their phosphorylation level is plotted here. In a paired t-test, p=0.08 whilst in an unpaired t test p=0.04 indicating the difference is probabably not significant.

Free wall samples n=4 measurements, mean phosphorylation level is 2.42±0.11 (SEM)

Septum samples n=13 measurements, mean phosphorylation level is 1.87±0.24

All samples n=17 measurements, mean phosphorylation level is 2.00±0.20
